# Prevalence of coronary artery disease and its risk factors in Kerala, South India: a community-based cross-sectional study

**DOI:** 10.1186/s12872-016-0189-3

**Published:** 2016-01-14

**Authors:** M. N. Krishnan, G. Zachariah, K. Venugopal, P. P. Mohanan, S. Harikrishnan, G. Sanjay, L. Jeyaseelan, K. R. Thankappan

**Affiliations:** Govt. Medical College, Kozhikode, Kerala India; Mother Hospital, Thrissur, Kerala India; Pushpagiri Hospital, Tiruvalla, Kottayam, Kerala India; Westfort High-tech Hospital, Thrissur, Kerala India; Sree Chitra Tirunal Institute for Medical Sciences and Technology, Trivandrum, Kerala India; Department of Biostatistics, Christian Medical College, Vellore, Tamil Nadu India; Achutha Menon Centre for Health Science Studies, Sree Chitra Tirunal Institute for Medical Sciences and Technology, Trivandrum Medical College, P.O. 695011 Thiruvananthapuram, Kerala India

**Keywords:** Coronary artery disease, Coronary risk factors, Prevalence, Kerala, India

## Abstract

**Background:**

There are no recent data on prevalence of coronary artery disease (CAD) in Indians. The last community based study from Kerala, the most advanced Indian state in epidemiological transition, was in 1993 that reported 1.4 % definite CAD prevalence. We studied the prevalence of CAD and its risk factors among adults in Kerala.

**Methods:**

In a community-based cross sectional study, we selected 5167 adults (mean age 51 years, men 40.1 %) using a multistage cluster sampling method. Information on socio-demographics, smoking, alcohol use, physical activity, dietary habits and personal history of hypertension, diabetes, and CAD was collected using a structured interview schedule. Anthropometry, blood pressure, electrocardiogram, and biochemical investigations were done using standard protocols. CAD and its risk factors were defined using standard criteria. Comparisons of age adjusted prevalence were done using two tailed proportion tests.

**Results:**

The overall age-adjusted prevalence of definite CAD was 3.5 %: men 4.8 %, women 2.6 % (*p* < 0.001). Prevalence of any CAD was 12.5 %: men 9.8 %, women 14.3 % (*p* < 0.001). There was no difference in definite CAD between urban and rural population. Physical inactivity was reported by 17.5 and 18 % reported family history of CAD. Other CAD risk factors detected in the study were: overweight or obese 59 %, abdominal obesity 57 %, hypertension 28 %, diabetes 15 %, high total cholesterol 52 % and low level of high density lipoprotein cholesterol 39 %. Current smoking was reported only be men (28 %).

**Conclusion:**

The prevalence of definite CAD in Kerala increased nearly three times since 1993 without any difference in urban and rural areas. Most risk factors of CAD were highly prevalent in the state. Both population and individual level approaches are warranted to address the high level of CAD risk factors to reduce the increasing prevalence of CAD in this population.

## Background

Coronary artery disease (CAD) is the foremost cause of disability and death the world over and is one of the top five causes of death in Indian population [1]. Mortality from CAD in Indians is predicted to increase rapidly and overtake that of the high-income countries. Among adults over 20 years of age, there has been a two-fold rise in CAD in rural areas and a 6-fold rise in urban areas during the period from 1960 to 2002 [2]. Previous studies have shown high prevalence of CAD in Asian Indians residing in the United States [3]. However there are no robust and contemporary data on CAD in native Indians. In a systematic review of CAD prevalence from India, Ahmed et al. commented that none of the studies conformed to the requirements of a high-quality epidemiologic study [4]. Kerala, with a population of over 33 million, is the most advanced state in epidemiological transition and has the highest prevalence of CAD risk factors in India [5]. The state has been reported to be the harbinger of what the rest of India is going to face in the near future [5]. The INTERHEART study reported the importance of conventional risk factors associated with CAD [6]. Although the CAD risk factor prevalence is the highest in the state of Kerala, there are no recent studies on the prevalence of CAD in this state. The only one community based study in 1993 from the rural area of the southernmost district of the state reported a CAD prevalence of 7.4 % [7]. The environment in Kerala is conducive for increasing the CAD risk factors [8] which is likely to result in an increase in the CAD prevalence. Therefore we wanted to study the current prevalence of CAD and its major risk factors in Kerala.

## Methods

**Fig. 1:**
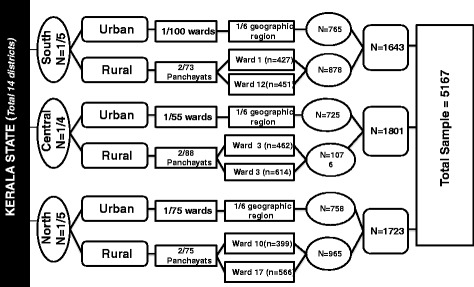
Sample selection procedure

A detailed description of the design, sample, and methods of the study has already been published [9]. Briefly, this was a cross-sectional community-based study during the period from January to June 2011. We estimated the sample size based on an anticipated prevalence of 7.4 % of CAD for rural Kerala based on the data by Kutty et al. [7] and 11 % for urban Kerala based on the study by Mohan et al. in Chennai [10]. The total sample size was estimated to be 3000 in rural and 2400 in urban areas. Sample selection procedure is given in Fig. 1. Subjects aged 20–79 years were selected using a multistage cluster sampling procedure. In the first stage three out of the 14 districts of Kerala state were selected. The 14 districts were grouped into five northern, five southern and four central districts. From the northern group of districts, Kozhikode district was selected which included the city corporation of Kozhikode. In the southern group of districts, Thiruvananthapuram and Kollam districts had city corporations. Of these Thiruvananthapuram was selected randomly. In the central group of districts there were two districts which included corporations; Ernakulam and Thrissur. Of these Thrissur district was selected randomly. In the second stage, one ward was randomly selected from each of the three city corporation wards in the urban area. Each of the selected wards was divided into six geographic regions. One of these geographic regions was selected randomly as the study area. All the households in this area were eligible to be included in this sample. One subject between 20 and 59 years was selected using KISH method [11] and all the subjects between 60 and 79 years were included.

In rural area, two of the Panchayats from the above three districts were randomly selected. From each of these Panchayats, one ward was randomly selected. All the households in the selected ward were eligible to be included on the sample. One subject between the age of 20 and 59 years was selected using KISH method and all subjects between 60 and 79 years were included.

Each selected individual was invited to visit an easily accessible facility with all past medical records after overnight fasting. Trained investigators collected all the relevant data.

Using a structured interview schedule, information on basic socio-economic and demographic details, smoking, physical activity, dietary habits, personal history of hypertension, dyslipidemia, diabetes mellitus and CAD were collected. The instrument also contained the Rose Angina Questionnaire (RAQ) [12, 13] and questions related to history of documented prior myocardial infarction, unstable angina, coronary artery bypass grafting (CABG) surgery, noninvasive investigations for CAD, coronary angiography, coronary angioplasty, documented use of drugs for CAD and hospital admission for CAD. Family history of ischemic heart disease, stroke and coronary risk factors was also recorded.

### Anthropometric measurements

Height was measured by wall-mounted stadiometer (Model 206, Seca, Hamburg Germany) to the nearest centimeter. Subjects were asked to stand upright without shoes, with their back against the wall, heels together and eyes directed forward.

Weight was measured with a portable electronic weighing scale (Model HN 283, Omron Corporation, Shimogyo-ku, Kyoto, Japan) kept on a firm horizontal surface. The subjects were asked to wear light clothing and remove footwear. Weight was recorded in kilograms to the nearest 0.5Kg. Body mass index was calculated as weight in kg/(height in meter squared) [14].

Waist circumference was measured using a non-stretchable measuring tape. The subjects were asked to stand erect in a relaxed position with both feet together. Waist girth was measured at the midpoint between the iliac crest and the lower margin of the ribs at the end of expiration, to the nearest centimeter.

### Blood pressure

Blood pressure was recorded with electronic apparatus (model 1A2, Omron Corporation, Shimogyo-ku, Kyoto, Japan) in sitting position, on the left arm resting on a table at heart level, after the subject having rested for at least 15 min. Three readings were taken 3 min apart and the mean of the last two readings was recorded as the BP. Heart rate was also recorded.

### Electrocardiogram

Resting 12 lead electrocardiogram (ECG) was performed on all subjects by trained technicians with three-channel digital ECG recorders with facility for display and measured parameters. For each lead, five consecutive complexes were recorded. Minnesota coding [15] and application of CAD criteria were performed by an experienced cardiologist for the respective region; those subjects diagnosed to have CAD were re-evaluated by another cardiologist in a blinded manner. Disagreements were resolved by consensus. Similar procedure was followed for 10 % of randomly selected subjects without a diagnosis of CAD.

### Biochemical investigations

Blood samples were drawn from individuals after 10–12 h of fasting. Plasma glucose was estimated immediately, onsite using the Glucose oxidase/ peroxidase- phenol-4-amenophenazone method (GOD-PAP). Blood samples for lipid profile measurement were transported on ice packs (4–6 °C) to an accredited core laboratory (Thyrocare Technologies Ltd, Navi Mumbai, India) on the same day. Estimation was carried out within 48 h in clinical chemistry instruments (Olympus AU2700) and Advia 1800 chemistry system (Siemens) using standard commercially available kits (Agappe Diagnostics). Photometry technology was used for lipid profile. Serum cholesterol was measured by cholesterol oxidase phenol 4- aminoantipyrine peroxidase (CHOD-PAP) method and serum triglycerides by glycerol-3 -phosphate oxidase -p - aminophenzone peroxidase (GPO-PAP) method. High density lipoprotein (HDL) cholesterol was estimated by enzyme selective protection method. The reaction between cholesterol assays is suppressed by the electrostatic interaction between polyanions & cationic substances. Low-density lipoprotein (LDL) cholesterol was calculated by using Friedwald’s equation.

### Ethical clearance

The present study was in compliance with the Helsinki Declaration. The study was approved by the Ethics Committee of Cardiological Society of India, Kerala Chapter. Informed written consent was obtained from all participants.

### Definitions used

We defined coronary artery disease as: (a) Definite CAD based on any of: documented evidence of prior acute coronary syndrome (ACS) or treatment for CAD, documented history of undergoing coronary angioplasty or CABG, more than 50 % epicardial coronary stenosis by invasive coronary angiography, ECG showing pathological Q waves (any of Minnesota code 1-1-1 to 1-1-7 or 1-2-1 to 1-2-5 or 1-2-7), imaging evidence of a region of loss of viable myocardium that is thinned and has a motion abnormality, in the absence of a non-ischemic cause [16], RAQ angina plus ECG changes (any of Minnesota codes 4-1-1, 4-1-2, 4-2 or 5-l, 5-2), or RAQ angina plus positive treadmill ECG (exercise-induced horizontal or down-sloping ST depression of ≥ 1 mm at 80 ms from J point), or inducible ischemia on stress imaging;(b) Probable CAD based on any of (in the absence of any of the definite criteria): RAQ angina without significant ECG changes, ECG changes (any of Minnesota Code 4-1-1,4-1-2, 4-2 or 5-1, 5-2) without RAQ angina, or positive treadmill ECG without RAQ angina. Any CAD was defined as those who satisfied either definite or probable CAD criteria.

We defined diabetes mellitus as fasting blood glucose value of ≥ 7 mmol/L and/or if there was current use of medications for diabetes [17], hypertension as blood pressure ≥140 mm of Hg systolic and/or ≥90 mm of Hg diastolic and/or currently on drugs for high blood pressure [18], and dyslipidemia as any of: serum total cholesterol ≥ 5.18 mmol/L, serum LDL cholesterol ≥ 3.37 mmol/L, serum HDL cholesterol <1.04 mmol/L in men or < 1.29 mmol/L in women, or serum triglycerides ≥1.69 mmol /L [19]. We categorized body mass index (BMI) as normal (18.0–22.9 kg/m^2^), overweight (23.0–24.9 kg/m^2^), or obesity (≥25 kg/m^2^). Abdominal obesity was defined as a waist circumference of ≥90 cm in men or ≥80 cm in women [20]. Physical activity levels were classified into sedentary and non-sedentary. All subjects reporting physical activity for at least 30 min a day for a minimum of 5 days a week (household activities involving physical effort, walking to and from work involving at least 30 min., manual workers, those performing leisure-time physical activity) were considered non-sedentary. All others were classified as sedentary [21]. We determined the socioeconomic status of the participants using the validated standard of living Index tool developed by National Family Health Survey [22]. We divided our entire sample into tertiles, based upon the index score. Participants in the upper tertile were classified as high socioeconomic group, those in the middle tertile as intermediate socioeconomic group and lower tertile as low socioeconomic group.

### Statistical analysis

We entered data into CS Pro software (the US Census Bureau) version 4 · 0 for Windows. Data cleaning and statistical analysis were performed using Stata (Stata Corp, Texas, USA) version 13 · 0 for Windows. All proportions were age-adjusted using WHO population data. Frequency distribution was done for categorical variables. Comparisons of age-adjusted prevalence between different categories were done using two-tailed proportion test. The difference in the age-adjusted prevalence and its 95 % confidence intervals are provided. *P* value <0 · 05 defined the level of statistical significance.

## Results

Of the total 6477 individuals contacted, we could evaluate 5167 (men 40.1 %); overall response rate was 79.8 % (men 75.0 %, women 83.3 %). There were no missing data in the interview schedule, ECG or anthropometrics. Data on blood sugar and serum cholesterol were missing in 32 and 47 subjects respectively; these were excluded from analysis.

Table 1 depicts the demographic and behavioural characteristics of the participants by area. There were greater proportion of participants without formal education in rural area as compared to urban; those with >10 years of education were more in urban as compared to rural area. The proportion of subjects that reported current smoking was similar in rural and urban men. There was larger proportion of vegetarianism, obesity and abdominal obesity among urban participants.

**Table 1:** Baseline demographic and behavioural characteristics of the participants by area

Variable		Urban		Rural		Total		
		(*N* = 2248)		(*N* = 2919)		(*N* = 5167)		
		n	(%)	n	(%)	n	(%)	*P* value
Age								
	20–29	111	4.94	225	7.71	336	6.50	<0.001
	30–39	345	15.35	543	18.60	888	17.19	
	40–49	507	22.55	692	23.71	1199	23.20	
	50–59	493	21.93	531	18.19	1024	19.82	
	60–69	566	25.18	650	22.27	1216	23.53	
	70–79	226	10.05	278	9.52	504	9.75	
Sex								
	Men	971	43.19	1101	37.72	2072	40.10	<0.001
	Women	1277	56.81	1818	62.28	3095	59.90	
Socio-economic status								
	Low	630	28.02	1303	44.64	1933	37.41	<0.001
	Middle	792	35.23	743	25.45	1535	29.71	
	High	826	36.74	873	29.91	1699	32.88	
Educational status								
	No formal education	87	3.87	203	6.95	290	5.61	<0.001
	1–4 years	208	9.25	437	14.97	645	12.48	
	5–10 years	1294	57.56	1729	59.23	3023	58.51	
	>10 years	659	29.31	550	18.84	1209	23.40	
Smoking^a^								
	Never	523	55.52	443	44.04	966	49.59	<0.001
	Past	111	11.78	220	21.87	331	16.99	
	Current	308	32.70	343	34.10	651	33.42	
Physical activity								
	Sedentary	1770	78.74	2328	79.75	4098	79.31	0.371
	Non-sedentary	478	21.26	591	20.25	1069	20.69	
Dietary habits								
	Vegetarian	161	7.16	110	3.77	271	5.24	<0.001
	Non-vegetarian	2087	92.84	2809	96.23	4896	94.76	
BMI								
	Low	111	4.95	244	8.37	355	6.88	<0.001
	Normal	685	30.53	1042	35.76	1727	33.48	
	Overweight	450	20.05	527	18.09	977	18.94	
	Obese	998	44.47	1101	37.78	2099	40.69	
Abdominal Obesity								
	Yes	1394	62.20	1709	58.67	3103	60.21	<0.001
	No	847	37.80	1204	41.33	2051	39.79	

^a^ Smoking in men only

### Prevalence of coronary artery disease

The crude and age-adjusted prevalence of definite CAD in Kerala was 5.8 and 3.5 % respectively. The figures for men were 8.0 % and 4.8 % and for women 4.2 and 2.6 % respectively; the differences were significant across gender (*p* < 0.001). The overall crude and age-adjusted prevalence of any CAD in Kerala was 16.6 and 12.5 % respectively. The crude and age-adjusted prevalence of any CAD in men were 14.6 and 9.8 % and in women were 17.9 and 14.3 % respectively; the prevalence was significantly higher in women as compared to men (*p* < 0.001). Table 2 outlines the prevalence of CAD (any & definite) among different age groups and gender. As age increased, the prevalence of CAD increased in both gender.

**Table 2:** Crude and age-adjusted prevalence of CAD by age group and gender

Age group (yrs.)	Men			Women			Difference (%)	95 % CI	*p* value
Any CAD	n	P (%)	SE	n	P (%)	SE			
20–29	144	2.78	1.37	192	7.29	1.88	−4.51	(−9.07,0.04)	0.069
30–39	306	6.54	1.42	582	8.93	1.18	−2.40	(−6.01,1.21)	0.213
40–49	465	7.10	1.19	734	14.44	1.30	−7.34	(−10.80,−3.89)	<0.001
50–59	386	12.44	1.68	638	19.91	1.58	−7.47	(−11.99,−2.95)	0.002
60–69	537	22.72	1.81	679	25.77	1.68	−3.05	(−7.89,1.78)	0.218
70–79	234	32.05	3.06	270	29.26	2.77	2.79	(−5.28,10.87)	0.497
All	2072	14.58	0.78	3095	17.87	0.69	−3.29	(−5.32,−1.26)	0.002
Age adjusted	2072	9.80	0.65	3095	14.26	0.69	−4.46	(−6.24,−2.69)	<0.001
Definite CAD									
20–29	144	0.00	0.00	192	0.00	0.00	-	-	-
30–39	306	2.29	0.86	582	0.52	0.30	1.77	(0.00,3.55)	0.017
40–49	465	3.87	0.90	734	1.77	0.49	2.10	(0.10,4.10)	0.026
50–59	386	6.48	1.25	638	3.13	0.69	3.34	(0.54,6.14)	0.012
60–69	537	13.41	1.47	679	8.98	1.10	4.42	(0.83,8.02)	0.014
70–79	234	18.80	2.56	270	12.59	2.02	6.21	(−0.17,12.59)	0.055
All	2072	8.01	0.60	3095	4.23	0.36	3.78	(2.41,5.15)	<0.001
Age adjusted	2072	4.80	0.39	3095	2.63	0.23	2.17	(1.09,3.25)	<0.001

*CAD* coronary artery disease, *P* prevalence, *SE* standard error

The prevalence of definite CAD was similar irrespective of region or religion. The prevalence of any CAD was lowest among Christians and highest among Muslims (10 vs.15.8 % *p* = 0 · 002). Of the three geographical regions of Kerala, Thirvananthapuram had lower prevalence of any CAD compared to Thrissur and Kozhikode (8.3 vs. 14.4 and 14.7 % *p* < 0.001).

Table 3 depicts the age-adjusted prevalence of CAD (any and definite) by gender, age group (age ≤ 45 and >45 years), and by area. Age-adjusted prevalence of any CAD in participants ≤45 years and >45 years was 7.8 and 18.7 % respectively. Similarly the age-adjusted prevalence of definite CAD was 0.9 % in participants ≤45 years and 6.8 % in >45 years. Age-adjusted prevalence of CAD (any and definite) was significantly higher in participants >45 years (*p* < 0.001). The prevalence of any CAD was significantly higher in rural women (15.6 %) as compared to urban women (11.7 %) (*p* < 0.01). The overall prevalence of any CAD was higher in rural (13.2 %) than urban (11 · 3 %) area (*p* = 0.038). However, there was no difference between urban and rural areas in the overall prevalence of definite CAD (3.4 vs. 3.6 %) (*p* = 0.72).

**Table 3:** Age-adjusted prevalence of CAD by gender, age, and area

	Gender	=/<45 years			>45 years			Difference (%)	95 % CI	*p* value
		n	P (%)	SE	n	P (%)	SE			
Any CAD	Men	728	5.12	0.82	1344	15.05	1.04	−9.92	(−12.42,−7.43)	<0.001
	Women	1239	9.48	0.92	1856	21.08	1.10	−11.60	(−14.07,−9.13)	<0.001
	All	1967	7.78	0.64	3200	18.68	0.78	−10.90	(−12.69,−9.10)	<0.001
Definite CAD	Men	728	1.82	0.43	1344	8.43	0.80	−6.61	(−8.39,−4.84)	<0.001
	Women	1239	0.36	0.15	1856	5.61	0.58	−5.25	(−6.35,−4.15)	<0.001
	All	1967	0.89	0.18	3200	6.80	0.47	−5.91	(−6.88,−4.95)	<0.001
		Urban			Rural					
Any CAD	Men	971	10.82	1.06	1101	8.94	0.81	1.88	(−0.70,4.46)	0.151
	Women	1277	11.66	0.88	1818	15.63	0.94	−3.97	(−6.40,−1.55)	0.002
	All	2248	11.31	0.68	2919	13.23	0.66	−1.92	(−3.72,−0.12)	0.038
Definite CAD	Men	971	4.92	0.57	1101	4.69	0.54	0.23	(−1.62,2.08)	0.806
	Women	1277	2.24	0.29	1818	2.84	0.33	−0.60	(−1.72,0.51)	0.299
	All	2248	3.38	0.29	2919	3.57	0.28	−0.18	(−1.19,0.82)	0.722

*CAD* coronary artery disease, *P* prevalence, *SE* standard error

Table 4 shows the age-adjusted prevalence of various criteria for diagnosis of CAD. ST code, T code, and RAQ angina were significantly higher in women; all other criteria were higher in men (*p* < 0.01). Table 5 represents the age-adjusted prevalence of various criteria for diagnosis of CAD among participants with any CAD. Again, most of the definite criteria for CAD were higher in men. Except ST code, T code, and RAQ angina, there was higher prevalence of various coronary artery criteria among men as compared to women (*p* < 0.001); ST code was higher in women, while T code and RAQ angina showed no significant difference. Among participants diagnosed to have any CAD, the prevalence of known CAD was 7.8 and 2.7 % in men and women respectively. Symptomatic CAD (sum of those with known CAD and RAQ angina) was prevalent in 54 % with any CAD.

**Table 4:** Age-adjusted prevalence of various CAD parameters among the total participant population

	Total		Men		Women		Difference (%)	95 % CI	*p* value
	*n* = 5167		*n* = 2072		*n* = 3095				
	P (%)	SE	P (%)	SE	P (%)	SE			
Q codes	0.87	0.11	1.65	0.24	0.32	0.08	1.33	(0.74,1.91)	<0.001
ST Code	1.87	0.19	1.00	0.18	2.46	0.29	−1.47	(−2.16,−0.77)	<0.001
T code	5.46	0.35	3.86	0.46	6.44	0.49	−2.59	(−3.79,−1.39)	<0.001
Documented ACS	1.59	0.14	2.70	0.30	0.84	0.13	1.86	(1.09,2.63)	<0.001
Documented treatment for CAD	2.26	0.16	3.55	0.34	1.39	0.16	2.17	(1.27,3.07)	<0.001
Angioplasty/CABG or Fibrinolysis	0.44	0.07	0.96	0.17	0.05	0.03	0.91	(0.48,1.34)	<0.001
Angiographic Coronary stenosis	0.63	0.09	1.15	0.19	0.27	0.07	0.88	(0.39,1.37)	<0.001
Imaging evidence of RWMA	0.06	0.09	1.34	0.20	0.10	0.05	1.24	(0.73,1.74)	<0.001
RAQ angina	6.38	0.35	4.71	0.44	7.47	0.51	−2.76	(−4.06,−1.46)	<0.001
Positive treadmill ECG	0.54	0.08	0.98	0.17	0.22	0.07	0.76	(0.30,1.21)	<0.001

**Table 5:** Age-adjusted prevalence of various CAD parameters among any CAD

	Total		Men		Women		Difference (%)	95 % CI	*p* value
	*n* = 855		*n* = 302		*n* = 553				
	P (%)	SE	P (%)	SE	P (%)	SE			
Q codes	4.88	0.79	12.22	2.48	1.49	0.40	10.74	(6.91,14.57)	<0.001
ST Code	12.94	2.16	7.08	1.51	15.14	2.74	−8.07	(−12.23,−3.91)	<0.001
T code	43.06	3.44	45.84	6.35	42.61	3.92	3.23	(−3.74,10.20)	0.3625
Documented ACS	8.68	0.98	20.36	2.85	3.80	0.71	16.56	(11.75,21.37)	<0.001
Documented treatment for CAD	11.88	1.05	26.56	2.95	5.98	0.79	20.59	(15.23,25.94)	<0.001
Angioplasty/CABG or Fibrinolysis	2.18	0.41	6.63	1.44	0.19	0.11	6.43	(3.60,9.26)	<0.001
Angiographic Coronary stenosis	3.21	0.55	8.07	1.81	1.10	0.31	6.98	(3.78,10.17)	<0.001
Imaging evidence of RWMA	3.03	0.46	8.75	1.56	0.40	0.19	8.34	(5.11,11.57)	<0.001
RAQ angina	49.69	3.44	43.98	6.43	51.60	3.96	−7.62	(−14.60,−0.64)	0.033
Positive treadmill ECG	2.74	0.52	6.26	1.33	1.12	0.51	5.14	(2.27,8.01)	<0.001

Q code-any of the Minnesota Q code 1-1-1 to 1-1-7 or 1-2-1 to 1-2-5 or 1-2-7; ST code-any of the Minnesota ST code 4-1-1,4-1-2 or 4–2; T code -any of the Minnesota T code 5-1 or 5-2; *ACS* acute coronary syndrome, *CABG* coronary artery bypass graft surgery, *RWMA* regional wall motion abnormality, *ECG* electrocardiogram, *P* prevalence, *SE* standard error

### Coronary risk factors

Age-adjusted prevalence of major risk factors of CAD by gender and area is presented in Table 6. Conventional risk factors like diabetes mellitus, hypertension, high serum cholesterol, low serum HDL cholesterol, smoking, physical inactivity, and family history of CAD were highly prevalent in the state. Except low HDL cholesterol (23.9 % in urban men vs. 27.6 % in rural men), all other major risk factors were higher in urban men as compared to rural men. This pattern was also observed in urban women as compared to rural women except high serum cholesterol (49.2 vs. 52.4 %; *p* = 0.089) and low HDL cholesterol (43.4 vs. 48.7 %; *p* =0.004).

**Table 6:** Age adjusted prevalence of major risk factors of CAD by gender and area

Risk factor	All			Men							Women							*p* value**
				Urban			Rural			*p* value*	Urban			Rural			*p* value*	
	n	%	SE	n	%	SE	n	%	SE		n	%	SE	n	%	SE		
Diabetes	5135	15.23	0.50	962	19.14	1.40	1097	16.23	1.16	0.0831	1272	15.40	0.95	1804	12.47	0.73	0.020	<0.001
Hypertension	5153	28.44	0.63	967	39.99	2.04	1095	26.24	1.33	<0.001	1275	30.15	1.29	1816	23.42	0.85	<0.001	<0.001
High cholesterol	5121	52.31	0.88	960	56.54	2.22	1092	53.36	1.85	0.1487	1264	49.23	1.76	1805	52.35	1.35	0.089	0.023
Low HDL	5120	38.55	0.88	959	23.92	1.94	1092	27.56	1.69	0.0603	1264	43.41	2.02	1805	48.73	1.44	0.004	<0.001
Smoking	2072	28.05	1.19	971	30.45	2.00	1101	26.88	1.48	0.0721	-	-	-	-	-	-	-	-
Physical inactivity	5167	17.45	0.64	971	30.72	2.06	1101	28.77	1.64	0.3315	1277	11.19	1.19	1818	9.38	0.64	0.101	<0.001
Family history	5167	18.35	0.64	971	18.97	1.47	1101	17.37	1.39	0.3467	1277	20.37	1.39	1818	16.92	1.02	0.015	0.939

*P* prevalence, *SE* standard error, *p value between urban and rural; **p value between men and women

Risk factor analysis of any and definite CAD is presented in Table 7. The age adjusted prevalence of risk factors such as hypertension, low HDL and family history were significantly higher in any CAD group (*p* value <0.01). However, higher cholesterol and smoking were higher in no CAD group. In definite CAD, all risk factors except low HDL were statistically significant (*p* value <0.01). In addition, excluding higher cholesterol all age adjusted prevalence of these conventional risk factors was higher in definite CAD as compared to no CAD.

**Table 7:** Risk factors analysis of any and definite CAD

Risk factor	Any CAD									Definite CAD								
	Yes			No			Difference (%)	95 % CI	*p* value	Yes			No			Difference (%)	95 % CI	*p* value
	n	%	SE	n	%	SE				n	%	SE	n	%	SE			
Diabetes	851	17.07	1.97	4284	14.89	0.52	2.18	(−0.56,4.93)	0.106	296	33.30	5.24	4839	14.73	0.50	18.57	(13.11,24.03)	<0.001
Hypertension	852	32.19	2.04	4301	27.67	0.67	4.51	(1.10,7.92)	0.008	295	51.32	5.34	4858	28.04	0.64	23.29	(17.44,29.13)	<0.001
High cholesterol	849	47.63	3.24	4272	52.82	0.92	−5.19	(−8.86,−1.51)	0.006	293	41.01	5.68	4828	53.01	0.88	−12.00	(−17.81,−6.20)	<0.001
Low HDL	849	43.62	3.53	4271	37.59	0.92	6.04	(2.40,9.68)	0.001	293	42.76	5.90	4827	38.33	0.89	4.44	(−1.39,10.26)	0.13
Smoking	855	6.91	1.02	4312	11.80	0.56	−4.89	(−6.85,−2.94)	<0.001	297	15.69	4.42	4870	11.22	0.53	4.46	(0.23,8.69)	0.019
Physical inactivity	855	15.55	2.17	4312	17.49	0.68	−1.94	(−4.62,0.74)	0.170	297	31.02	5.32	4870	17.13	0.65	13.89	(8.52,19.25)	<0.001
Family history	855	27.06	3.09	4312	17.37	0.66	9.70	(6.51,12.88)	<0.001	297	25.29	4.06	4870	18.03	0.65	7.27	(2.21,12.33)	0.002

## Discussion

### Prevalence of coronary artery disease

Coronary artery disease has been gaining importance as a major public health problem in India. In 2003, the prevalence of CAD was estimated to be 3–4 % in rural areas and 8–10 % in urban areas according to a population-based cross sectional survey [23]. Our study also showed high prevalence of CAD in Kerala. The age-adjusted overall prevalence of definite CAD was 3.5 % and any CAD was 12.5 %. We chose criteria for any CAD to detect all possible cases of CAD; for definite CAD we have avoided less specific criteria like RAQ angina, positive treadmill electrocardiogram or minor electrocardiographic changes in isolation and used combined criteria when these less specific components were incorporated.

Age-adjusted prevalence of definite CAD was higher in men. However, any CAD prevalence in women was significantly higher than in men. It is well known that women commonly have ECG with nonspecific ST –T changes and angina with normal coronary arteries; this may have resulted in overestimation of any CAD prevalence in women. In our study the prevalence of RAQ angina and ST-T changes were significantly higher among women whose proportion in rural area (62 %) was higher than that of urban area (57 %). Earlier population studies also consistently recorded much higher prevalence of CAD in women using RAQ angina and ST-T criteria for diagnosis of CAD [24–27]. It has also been noted that RAQ angina is less reliable in women [28] and non-white population [29]. Moreover, Patel et al. in a study from migrant South Asians observed that the value of ST-T changes alone as indicator of CAD was questionable, especially in women [30].

This study showed no difference in the prevalence of definite CAD between urban and rural population of Kerala while any CAD was slightly more in the rural population. Previous studies from other parts of India have highlighted the marked urban preponderance of CAD [25, 31, 32]. Some of the risk factors such as general obesity and abdominal obesity were higher in urban areas, whereas smoking was higher in rural area. There was no difference in physical inactivity in rural and urban areas. In most other Indian states there are huge differences between urban and rural areas. However, in Kerala these differences are either minimal or some risk factors are higher in urban while others are higher in rural as noted above. This could be the reason for not having significant difference of definite CAD prevalence in urban and rural areas of Kerala.

The overall age-adjusted prevalence of any CAD in subjects at or below the age of 45 years was 7.8 %. Our study showed a higher prevalence of CAD among young subjects compared to a previous study [7]; in this 1993 study, there were no subjects with definite CAD below the age of 45 years where as we found definite CAD in 0.9 %. It is well known that coronary artery disease occurs in Indians 5–10 years earlier [33, 34]. The average age of first myocardial infarction in Asians is 53 years; among the three Asian populations, Chinese, Malay, and Indian, the highest age-adjusted incidence of coronary events in both sexes was in Indians [6]. The age-adjusted prevalence of CAD in young subjects in our study was similar to the US data on prevalence of CAD in the young (1.2 %).

Comparisons with previous Indian studies on CAD prevalence may be problematic due to heterogeneity in period of study, sample characteristics, and criteria for diagnosis of CAD. We did a systematic search for articles in PubMed and EMBASE published during the period from 1990 to 2014 in English language on the prevalence of CAD in India by community-based surveys. We chose only studies that had a minimum sample size of 1000, and that based the diagnosis on documented history of CAD and ECG criteria. We could collect 11original articles, one systematic review [4] and one meta-analysis [35]. The studies were heterogeneous in terms of sampling methods, subject groups, and criteria for diagnosis. Most of the studies were from northern India. Only one study mentioned the period of field survey [25]. Only one study analyzed the data for definite CAD and probable CAD separately [7]. Most studies were on either rural or urban population; only four covered both the groups [25, 31, 32, 36]. Sample size calculations were reported in three studies [7, 25, 31]. In a study from Delhi during the period from 1984 to 1987, Chadda et al. reported unadjusted prevalence of 9.7 % in urban and 2.7 % in rural inhabitants [25]. The sample consisted of men and women aged 24–64 years; they used clinical history of CAD and electrocardiographic criteria for diagnosis of CAD. Gupta et al. published an epidemiological survey of urban inhabitants over 20 years of age in Rajasthan, India [27]. The prevalence of CAD was 7.6 % (6 % in men and 10.4 % in women). The age distribution of the sample was consistent with that of the population. Singh et al. in 1997 surveyed an urban and rural sample of 3575 subjects between the ages 25 and 64 years from North India and reported CAD prevalence of 9 and 3.3 % respectively [31]. Mohan and colleagues reported 9 % age-adjusted prevalence of CAD in an urban sample of Chennai, South India [10]. More recently, another study from India noted 12.6 % crude CAD prevalence in an urban population [37]. All these studies had used less specific criteria for diagnosis of CAD; definite CAD as we had analyzed had not been computed in these studies. A study from Pakistan showed a crude prevalence of definite CAD in 6.1 men and 4 % women; they found ischemic ECG changes much more prevalent in women [38].

The US statistics reported 6 % age-adjusted CAD prevalence in respondents above 18 years by survey based on self-reported CAD by telephonic interview [39]. The prevalence between ages 18–44 years was 1.2 %. The update on heart disease and stroke in the US reported CAD prevalence of 6.4%in adults ≥ 20 years of age. The prevalence of CAD was 7.9 for men and 5.1 % for women [40]. The higher figure in the US data may be due to differences in age, sample, survey methods, and criteria for diagnosis. The reported prevalence of CAD in the United Kingdom was 3.5 % [41]; this figure was similar to our data on definite CAD.

As to the figures for definite CAD, only one study [7] reported the prevalence (1.4 %); they found no participant below the age of 45 years with definite CAD. However, it should be noted that the age range in this study was 25–64 years and the population was rural only. Compared to this, our data show an increase in the prevalence of definite CAD (0.9 %). Any CAD prevalence of 11.3 % in urban and 13.2 % in rural area respectively in our study was higher compared to some previous studies in the same age group and similar definition for CAD (8.1–9 % for urban and 3.5–5 % for rural) [10, 27, 42, 43]. These data indicate an increase in the prevalence of any CAD over the past two decades. The increase in prevalence of any CAD may partially be due to improvement of treatment of CAD thus allowing better survival.

The urban prevalence data in this study may be comparable to other cities of India; however, the rural figures for prevalence may not be comparable to most rural areas in rest of India since rural Kerala is different from rest of rural India in social and economic development.

### Prevalence of coronary risk factors

We found high prevalence of major conventional risk factors for CAD in our survey. The prevalence of various risk factors in our study like diabetes (15.2 %), hypertension (28.4 %), high cholesterol (52.3 %) and smoking in men (28.1 %) was comparable to the figures in a recent large study from Kerala, by Thankappan et al. [5]. The figures were also similar in the US adult population [44]. Physical activity in our study seems to be an overestimate due to the problems of self reports as has been reported by a recent study from Kerala [45].

### Limitations of the study

Our study was the largest and most comprehensive contemporary community-based survey on the prevalence of CAD and its risk factors from India. The geographic spread of the survey enabled us to derive meaningful inferences of prevalence from the state. We could capture all the necessary data from participants with very little missing variables. One of the limitations for our study was the sub-optimal response rate, which could reach only ~ 80 %; the response rate was lower in rural men. We could not capture the reasons for the poor response in men although we surmise that, being manual laborers many of them would be working on Sundays. As a result the sample consisted of higher proportion of women than in the general population. Sex disaggregated data will partly take care of this problem. There was significantly higher proportion of older individuals compared to the population of Kerala although age-adjustment in analysis would have removed this bias.

## Conclusion

Our study revealed a high age-adjusted prevalence of CAD and coronary risk factors in Kerala, South India. Our study did not show any difference in the CAD prevalence in urban and rural population. Our data on prevalence of CAD are comparable to the figures from the US and the United Kingdom. Compared to previous Indian studies and the 1993 study of Kerala, this study showed higher prevalence of CAD in Kerala. The prevalence of definite CAD in Kerala increased nearly three times since 1993 without any difference in urban and rural areas. Most risk factors of CAD were highly prevalent in the state. Both population and individual level approaches are warranted to address the high level of CAD risk factors to reduce the increasing prevalence of CAD in this population.

## Abbreviations

ACS: acute coronary syndrome
CHOD-PAP: cholesterol oxidase phenol 4-aminoantipyrine peroxidase
CABG: coronary artery bypass grafting
CAD: coronary artery disease
ECG: electrocardiogram
GOD-PAP: glucose oxidase/ peroxidase-phenol-4-amenophenazone method
HDL: high density lipoprotein
LDL): low-density lipoprotein
RAQ: rose angina questionnaire
